# Integrated Analysis of the ceRNA Network and M-7474 Function in Testosterone-Mediated Fat Deposition in Pigs

**DOI:** 10.3390/genes13040668

**Published:** 2022-04-10

**Authors:** Ximing Liu, Ying Bai, Ran Cui, Shuaihan He, Yao Ling, Changxin Wu, Meiying Fang

**Affiliations:** 1 National Engineering Laboratory for Animal Breeding, MOA Laboratory of Animal Genetics and Breeding, Department of Animal Genetics and Breeding, College of Animal Science and Technology, China Agricultural University, Beijing 100193, China; liuximing@cau.edu.cn (X.L.); ranc@cau.edu.cn (R.C.); heshuaihan@cau.edu.cn (S.H.); lingzi@cau.edu.cn (Y.L.); chxwu@cau.edu.cn (C.W.); 2 College of Life Sciences and Food Engineering, Hebei University of Engineering, Handan 056021, China; baiy1986@cau.edu.cn; 3 Sanya Institute of China Agricultural University, Sanya 572025, China

**Keywords:** ceRNA, castration, lncRNA, fat deposition, testosterone

## Abstract

Castration can significantly enhance fat deposition in pigs, and the molecular mechanism of fat deposition caused by castration and its influence on fat deposition in different parts of pigs remain unclear. RNA-seq was performed on adipose tissue from different parts of castrated and intact Yorkshire pigs. Different ceRNA networks were constructed for different fat parts. GO and KEGG pathway annotations suggested that testosterone elevates cell migration and affects differentiation and apoptosis in back fat, while it predisposes animals to glycolipid metabolism disorders and increases the expression of inflammatory cytokines in abdominal fat. The interaction between M-7474, novel_miR_243 and *SGK1* was verified by dual fluorescence experiments. This ceRNA relationship has also been demonstrated in porcine preadipocytes. Overexpression of M-7474 significantly inhibited the differentiation of preadipocytes compared to the control group. When 100 nM testosterone was added during preadipocyte differentiation, the expression of M-7474 was increased, and preadipocyte differentiation was significantly inhibited. Testosterone can affect preadipocyte differentiation by upregulating the expression of M-7474, sponging novel-miR-243, and regulating the expression of genes such as SGK1. At the same time, HSD11B1 and SLC2A4 may also be regulated by the corresponding lncRNA and miRNA, which ultimately affects glucose uptake by adipocytes and leads to obesity.

## 1. Introduction

Epidemiological data show that the testosterone level of middle-aged and elderly men gradually decreases with age, while the body fat content obviously increases [1]. It is believed that the sex hormones (mainly testosterone) secreted by the male gonads affect the growth and development of muscle, bone, fat and carcass quality [2,3,4]. The predominant and most active androgen is testosterone, which is produced by the male testes [5]. Animal experiments show that testosterone deficiency can increase fat accumulation in mice, leading to obesity [6]. Serum testosterone levels were significantly higher (*p* < 0.01), and fat content was significantly lower in intact boars than in barrows [7]. Castration affects the chemical composition of muscles, increasing the fat content and decreasing the water content [8,9]. A previous experiment in our group also revealed that castration promoted fat deposition and obesity in pigs fed a normal diet [10], which indicates that castration-induced obesity is significantly associated with testosterone deficiency.

Testosterone plays a pivotal role in the regulation of body fat distribution in animals [11]. Adipose tissue is an important site of androgen action, and testosterone can affect adipocyte proliferation and differentiation, thereby affecting body fat fraction adipocyte function and lipid metabolism [12]. Adipose tissue has been identified as an endocrine organ secreting adipokines involved in metabolic and inflammatory pathways, and testosterone plays an extremely important role in fat metabolism [13]. The distribution area of adipose tissue is closely related to disorders of glucose metabolism and lipid metabolism. There are obvious differences in phenotypic structure, metabolic pathway and function between visceral adipose tissue and subcutaneous adipose tissue deposited under the back. Visceral adipocytes have a higher fat content, a wider distribution of blood vessels and nerves, a weaker preadipogenic differentiation ability, and a higher proportion of mature adipocytes [14]. At the same time, due to higher levels of adrenocortical hormone and androgen receptors in visceral fat [15], the fat catabolism activity, insulin resistance and glucose uptake capacity are higher than those in subcutaneous fat [16], which is more sensitive to testosterone stimulation. The absorption capacity of subcutaneous fat for circulating free fatty acids and triglycerides is stronger [16]. Therefore, it is particularly important to study the regulatory factors of adipose tissue aggregation and distribution to control the development of obesity. The regulation of hormones, especially testosterone, with respect to the accumulation and distribution of adipose tissue has attracted increasing attention from researchers.

Long noncoding RNAs (lncRNAs) are noncoding RNAs with a length of >200 nt that can regulate the expression of target genes at the levels of transcription, posttranscription and apparent modification and thus regulate life activities. In recent years, research on lncRNAs has gradually increased, and their scope of action has been revealed to cover almost every aspect of life activity. Some lncRNAs have been shown to play an important regulatory role in the process of fat development: for example, brown fat lncRNA1 (Blnc1) can regulate the differentiation and thermogenic processes of brown and beige adipose tissues by forming a feedback loop with the transcription factor EBF2. This complex can also work with early B-cell factor 2 (EBF2) to enhance the expression of thermogenic genes, such as uncoupling protein 1 (UCP1) [17]. Lnc-BATE 1 is a regulatory factor needed for brown adipose tissue formation and thermogenesis by combining with heteronuclear ribonucleoprotein U (hnRN-PU) to produce the regulatory factors required for thermogenesis; ncRNA SRA can bind to peroxisome proliferator-activated receptor γ (PPARγ) and enhance PPARγ activity. There are many other ways to promote the differentiation of preadipocytes into adipocytes and further regulate the function of fat [18,19,20]. However, the molecular mechanisms of lncRNAs involved in fat deposition after castration in pigs remain unclear.

Although some studies [10,21,22,23] have shown that castration can significantly enhance fat deposition in pigs, the molecular mechanisms of fat deposition caused by castration and its influence on fat deposition in different parts of pigs are still unknown. Our research group compared 180-day-old whole-sib-derived castrated pigs with intact pigs and found that the backfat thickness and abdominal fat weight of castrated pigs were significantly increased. This study established an animal model of testosterone deficiency regulating pig obesity and observed the effects of castration on fat accumulation in the back and abdomen of Yorkshire pigs. The present study aimed to comprehensively investigate the alterations in the expression levels of long noncoding RNA (lncRNA), microRNA (miRNA/miR) and mRNA in adipose tissue in different parts of Yorkshire pigs after castration to provide a reference for elucidating the molecular mechanisms by which testosterone affects adipose deposition and the specific mechanisms by which testosterone affects fat deposition in different parts by constructing a ceRNA network. Our main purpose is to explore the effects of testosterone on fat deposition in pigs, which affects meat quality as well as pig production benefits. It also provides reference for obesity caused by testosterone deficiency in humans.

## 2. Materials and Methods

### 2.1. Animal Sample Collection and RNA Isolation

#### Tissue Samples

In this experiment, ten full-sibling male Yorkshire piglets were selected and divided into five pairs according to the principle of pairing design. The initial conditions of the test individuals were kept consistent as much as possible, and each pair of male piglets was composed of two full-sib individuals from the same source in the same litter and with similar body weights. At one week old, one piglet was randomly selected from each pair for surgical castration, and the other noncastrated individual was subjected to sham treatment (i.e., an incision with the same size as the castration operation was made in the abdomen on the premise of not damaging the gonads to produce the same stress effect) as the control. All pigs were bred under the same conditions, with free access to water and feed. All pigs were slaughtered, and phenotypes were recorded at 180 days of age. Three each of the castration and complete groups were used as biological replicates, and the same tissues were selected for RNA extraction and RNA-seq. The backfat was collected from the 6th–7th ribs of castrated and intact male pigs. Their suet was also collected as abdominal fat tissue. All tissue samples were frozen in liquid nitrogen for further use. There were significant differences in backfat thickness and abdominal fat weight between the castrated and intact males (*p* < 0.05) (Supplementary Materials File S3 Figure S1). More details about phenotype information are described in Supplementary Materials File S1 Table S1. Yorkshire pigs were raised by Beijing Zhongyu Pig Co., Ltd. (Beijing, China).

Total RNA was isolated using TRIzol Reagent (Invitrogen, San Diego, CA, USA) according to the manufacturer’s instructions. RNA quality was assessed using 1% agarose gels. RNA purity was determined using a K5500 Spectrophotometer (Kaiao, Beijing, China). RNA integrity and concentration were assessed using the RNA Nano 6000 Assay Kit and the Bioanalyzer 2100 system (Agilent Technologies, Foster, CA, USA).

### 2.2. Library Preparation for Long Noncoding RNA Sequencing and Data Analysis

Sequencing libraries were prepared using 3 µg of RNA per sample. Total RNA removes ribosomal RNA using an Epicenter Ribo-Zero Gold Kit (Epicenter, Madison, WI, USA) to retain all ncRNA to the maximum extent, randomly breaking the obtained product into short segments, synthesizing a cDNA first chain by using a sequence after the segments as a template by using a random hexamer (random hexamers), and adding a buffer solution, dNTPs, RNase H and DNA polymerase I to synthesize a cDNA second chain. The second strand was purified by a QIAquick PCR kit (Qiagen, Chatsworth, CA, USA) and eluted with EB buffer after end repair, the addition of base A, and the addition of sequencing linker, and then degraded by UNG (uracil-N-glycosylation) enzyme. Then, the fragment size was selected by agarose gel electrophoresis, and PCR amplification was performed. Products were purified using the AMPure XP system, and library quality was assessed with an Agilent Bioanalyzer 2100 system (Agilent Technologies, Santa Clara, CA, USA). The last constructed sequencing library was sequenced using Illumina HiSeq4000 (Illumina, San Diego, CA, USA), and 150 bp paired-end reads were generated. Raw data (raw reads) were processed using Perl scripts to remove reads containing an adapter, reads containing poly-N and reads of low quality. A low-quality read was defined as one in which more than 15% of bases had Phred quality scores less than or equal to 19. The remaining reads were mapped to the porcine reference genome (Sscrofa 11.1, Ensembl, ftp://ftp.ensembl.org/pub/release-92/fasta/sus_scrofa/dna/, accessed on 18 January 2020) using HiSAT2 (http://ccb.jhu.edu/software/hisat2/index.shtml, accessed on 18 January 2020) [24]. Mapped reads from each adipose tissue sample were assembled using StringTie [25] in a reference-based approach. After this, we evaluated the assembled transcripts using five criteria to identify lncRNAs: (1) transcripts with exon number <2 were removed; (2) transcripts with length ≤ 200 bp were removed; (3) known nonlncRNA annotations were removed; (4) transcripts with Fragments Per Kilobase of exon per Million fragments mapped (FPKM) < 0.5 were removed; (5) coding-noncoding-index (CNCI) v2 [26], coding potential calculator (CPC) 0.9-r2 [27], PFAM-scan v1.3 [28], and coding potential assessment tool (CPAT) [29] were used to distinguish mRNAs from lncRNAs. Transcripts predicted to have coding potential by all four tools described above were excluded, and those without coding potential were classified as lncRNA candidates. The transcripts excluded above were used as candidate mRNAs. PHAST v1.3 [30] was used for conservation analysis for coding genes and lncRNAs [31].

DESeq2 [32], EBSeq [33], NOISeq [34] and PoissionDis were used for differential gene expression analysis of lncRNAs and mRNAs between intact and castrated adipose tissue. LncRNAs and mRNAs with *p* < 0.05 and |log2 fold change| > 1 were identified as being differentially expressed between the two groups. We searched for potential cis targets (i.e., coding genes) 100 kb upstream and downstream from each lncRNA. Potential trans targets were identified by examining RNA data for coordinated expression using Pearson’s correlation coefficients (r > 0.90 or r < −0.90) as a classifier.

### 2.3. Library Preparation for Micro RNA Sequencing and Data Analysis

Three micrograms of RNA per sample was used as the input material for the small RNA library. Sequencing libraries were generated using the NEBNext Multiplex Small RNA Library Prep Set for Illumina (NEB, Ispawich, MA, USA) following the manufacturer’s recommendations. The library preparations were sequenced on a HiSeq 2500/2000 platform (Illumina), and 50 bp single-end reads were generated. Raw data (raw reads) were processed with Python scripts to remove defective reads. Clean reads with lengths in the desired range were used in all downstream analyses. The small RNAs were mapped to the porcine reference genome (Sus scrofa 11.1) using Bowtie [35] (no mismatches permitted) to analyze the expression and genomic distribution. Bedtools (https://bedtools.readthedocs.io/, accessed on 18 January 2020) was used to search for known miRNAs by matching them to entries in miRBase21 (http://www.miRbase.org/ftp.shtml, accessed on 18 January 2020). After excluding reads that were mapped to known miRNAs, miRDeep2 [36] was used to analyze the remaining reads to predict novel miRNAs. The prediction of miRNA target genes was performed using miRanda [37]. The levels of miRNA expression were estimated by RPM (reads per million total reads). Differential expression of miRNA between samples was analyzed using DEGseq2. miRNAs with significant differences (*p* < 0.05) and |log2 fold change| > 1 were classified as differentially expressed (DE) miRNAs.

### 2.4. Gene Ontology and Kyoto Encyclopedia of Genes and Genomes Enrichment Analysis

Gene ontology enrichment analysis of differentially expressed genes (DEGs) or DE lncRNAs and DE miRNA target genes was conducted using the GO-seq R package [38], correcting for gene length bias. GO terms with *p* < 0.05 were considered to be significantly enriched among DEGs. We used KOBAS v3.0 (http://kobas.cbi.pku.edu.cn/, accessed on 18 January 2020) [39] to test the statistical significance for the enrichment of DEGs or targets of DE lncRNAs and DE miRNAs in KEGG pathways.

### 2.5. Construction of LncRNA–miRNA–Gene Regulatory Networks

To construct the lncRNA–miRNA–target gene network, we first used BLASTN (https://blast.ncbi.nlm.nih.gov/, accessed on 18 January 2020) to identify and remove pre-microRNAs based on high levels of homology. Subsequently, miRanda was used to predict the target relationships between miRNAs and lncRNAs (we required an alignment score N = 160 and minimum free energy of −20 kcal/mol). As a competing endogenous RNA (ceRNA), a lncRNA can competitively bind miRNA with mRNA.

lncRNA–miRNA–gene pairs were further analyzed based on the common miRNA-binding sites [40]. We used miRanda and TargetScan (http://www.targetscan.org/, accessed on 18 January 2020) [41] to screen lncRNAs and genes for MRE sequence motifs and then constructed a lncRNA–miRNA–gene interaction network. The lncRNA, miRNA and mRNA interactions were constructed and visualized using Cytoscape v3.6.1 [42].

### 2.6. Quantitative Polymerase Chain Reaction

To detect DE lncRNAs and DEGs, total RNA (1 µg) from adipose tissue was transcribed into cDNA using the Fast Quant RT Kit (with gDNase) (Tiangen Biotech Co., Ltd., Beijing, China) according to the manufacturer’s instructions. After detecting DE miRNAs, total RNA (1 µg) from adipose tissue was transcribed into cDNA using the TaqMan MicroRNA Reverse Transcription Kit (Applied Biosystems, Foster City, CA, USA) according to the manufacturer’s instructions. The expression levels of six lncRNAs, six genes and six miRNAs were quantified with quantitative PCR (qPCR) using SYBR Green Real-time PCR Master Mix (Tiangen Biotech Co., Ltd., Beijing, China). Gene and lncRNA primers for qPCR were designed using Primer Premier 5.0 (Premier Biosoft International, Palo Alto, CA, USA) and were subsequently synthesized (Sangon Biotech, Beijing, China). MiRNA primers were designed and synthesized by RiboBio (Guangzhou, China). Primer sequences are listed in Supplementary Materials File S1 Table S2. The cycling parameters used for qPCR amplification were as follows: initial heat denaturation at 95 °C for 15 min, 40 cycles at 95 °C for 30 s, 60 °C for 30 s and 72 °C for 30 s, and a final extension at 72 °C for 5 min. A melting curve analysis was performed to exclude genomic DNA contamination and to confirm primer specificities. Gene and lncRNA expression levels were normalized using the 2^−ΔΔCt^ method with β-Actin (the expression of β-Actin was identified as stable in backfat and abdominal samples by semiquantitative reverse transcription (RT)-PCR) as an internal standard. Relative miRNA expression was normalized using the 2^−ΔΔCt^ method with U6 small nuclear RNA as an internal control. Each biological duplicate consisted of three technical replicates.

### 2.7. Vector Construction

Vectors required for double fluorescence experiments and preadipocyte differentiation experiments were constructed.

According to the sequence of targeted binding sites, psiCHECK-2 vectors (Promega, Madison, WI, USA) containing targeted binding sites (wild type) and mutation sites (mutant type) were synthesized by Qingke (Beijing, China). For example, M-7474 wild-type (p-M-7474) and mutant (p-mut-M-7474), *SGK1* wild-type (p-*SGK1*) and mutant (p-mut-*SGK1*) reporter vectors were constructed. The miRNA mimic NC was synthesized by Qingke (Beijing, China). The sequences of those vectors were as follows: novel-miR-243 mimic 5′ CGGGGAGGCUGUGCAGCGCGGCC 3′.

To stabilize the overexpression of M-7474 in porcine preadipocytes, according to the data obtained by our sequencing, the PCDH (YouBao, Chongqing, China) overexpression vector containing the full-length fragment of M-7474 was synthesized by 100 nmol vectors and transfected into cells using Lipofectamine 2000 (Invitrogen, 81 Wyman Street, Waltham, MA, USA).

### 2.8. Dual-Luciferase Reporter Analysis

HEK293T, a human cell line of renal epithelial cells, was used to validate the miRNA target. Cells were seeded into 24-well plates. Cotransfection with 200 ng of target mRNA-WT or target mRNA-MUT and 10 μL of miRNA mimic or mimic-NC was performed using Lipofectamine 2000 (Invitrogen). Subsequently, luciferase activity was measured using the Dual-Luciferase Reporter Assay System (Promega, Madison, WI, USA) at 48 h posttransfection. The assays were performed in triplicate.

293T cells were purchased from the Cell Bank of the Chinese Academy of Sciences and cultured in RPMI-1640 (Gibco, Grand Island, NY, USA) supplemented with 10% fetal bovine serum (FBS; Gibco), 100 units/mL penicillin (Gibco) and 100 mg/mL streptomycin (Gibco) at 37 °C in an incubator containing 5% CO_2_ and 95% air.

### 2.9. Overexpression and Differentiation of Preadipocytes

Preadipocytes were obtained from the Jingdong Yin group. After growing to 7 days of age, Yorkshire boars were slaughtered and primary preadipocytes were isolated from dorsal adipose tissue. Preadipocytes were cultured in DMEM/F12 (HyClone, Logan, UT, USA) supplemented with 10% fetal bovine serum (FBS) (Gibco), 100 mg/mL streptomycin (Life Technologies, Waltham, MA, USA) and 100 U/mL penicillin (Invitrogen).

To induce differentiation, primary preadipocytes were inoculated in 6-well plates until cell fusion. Subsequently, the cells were cultured in a medium containing 1 μM dexamethasone (Sigma, Ronkonkoma, NY, USA), 0.25 mM IBMX (Sigma), and 50 μg/mL insulin (Sigma). Culture medium was changed every 2 days, and the cells were frozen for future studies. The cells were further incubated for 48 h, after which the medium was replaced with maintenance medium (growth medium supplemented with 50 μg/mL insulin) and incubated for an additional 48 h. Then, the cells were cultured in growth medium until maturation at 6 days.

Based on previous studies from our laboratory [43], 100 nM testosterone was used to measure the effects on genes involved in preadipocyte differentiation. Testosterone (Sigma) (0.288 g) was dissolved in 10 mL methanol (Jingke, Changsha, China) to create a 1 mg/mL stock solution. In the process of preadipocyte differentiation, 20 µL of storage solution was added to 19.98 mL of differentiation medium to make a working solution with a concentration of 100 nM.

All preadipocyte experiments were performed in 6-well plates, and each treatment had three biological replicates. Four control experiments were designed by varying the testosterone content of the preadipocyte differentiation medium, including M-7474 + NC, NC + NC, M-7474 + 100 nM, and NC + 100 nM. In parallel, induced differentiation experiments in preadipocytes were performed, and cells were collected on day 6 of differentiation for relevant gene quantification and oil red O staining.

### 2.10. Statistical Analysis

Data are expressed as the means ± standard deviation (SD). Significance was analyzed using one-way analysis of variance (ANOVA) to test homogeneity of variances via Levene’s test, followed by Student’s *t* test. Calculations were conducted using SAS version 9.0 (SAS, Cary, NC, USA). Differences were considered to be statistically significant at *p* < 0.05.

## 3. Results

### 3.1. Overview of the Fat Deposition-Related Long Noncoding RNA, Messenger RNA and microRNA Transcription Profiles

**Figure 1:**
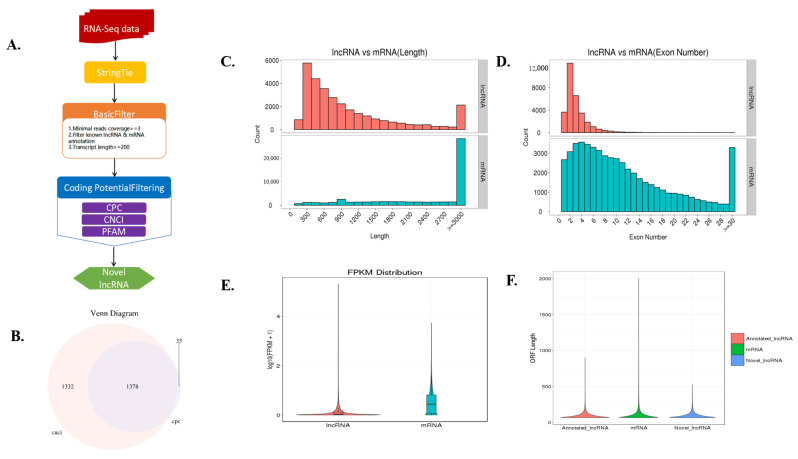
Identification of long noncoding RNAs (lncRNAs) and their comparison with mRNAs at the genomic structure and expression levels. (**A**) Workflow for lncRNA identification. (**B**) Candidate lncRNAs were identified by using two applications: CNCI (coding-noncoding-index) and CPC (coding potential calculator), which detect and remove putative protein-coding transcripts. (**C**) Distribution of lengths of lncRNAs and mRNAs. (**D**) Distribution of the number of exons of lncRNAs and mRNAs. (**E**) Expression levels of lncRNAs and mRNAs, calculated as log10(FPKM + 1). FRKM: Fragments per kilobase of exon per million fragments mapped. (**F**) The ORF sequence is converted to the length of the protein sequence.

The lncRNA libraries generated a total of 96–97.5 million remaining reads (clean reads) after excluding lower quality data. Approximately 80% of the clean reads were mapped to the reference genome (Supplementary Materials File S1 Table S3), and the correlation coefficients of gene expression for the three biological replicates in each group were greater than 0.80 (Supplementary Materials File S3 Figure S2). After additional filtering (Figure 1A) and removal of potential coding transcripts that were identified using CNCI and CPC (Figure 1B), a total of 1378 lncRNAs and 22,990 mRNAs were obtained. The predicted lncRNA molecules met the general characteristics of lncRNAs. lncRNAs were typically shorter than mRNAs (Figure 1C) and tended to contain only two or three exons, in contrast to the mRNAs (Figure 1D). lncRNAs also appeared to be expressed at lower levels than mRNAs (Figure 1E). The obtained ORF sequence was converted into a protein sequence, and the length distribution diagram is shown in Figure 1F.

miRNA libraries were analyzed. More than 23,004,493 (71.04%) clean reads in each sample were aligned with pig reference sequences. Most reads were 18–25 nt in length, which was the expected size for miRNAs. The MiRBase database was used to compare the miRNA information of pigs. The statistical data of known miRNAs in each sample are shown (Supplementary Materials File S2 Table S1). A total of 331 annotated mature miRNAs were identified (Supplementary Materials File S2 Table S2), while 708 novel mature miRNAs were identified (Supplementary Materials File S2 Table S3).

### 3.2. Differentially Expressed mRNAs and lncRNAs between Castrated and Intact Male Pigs

**Figure 2:**
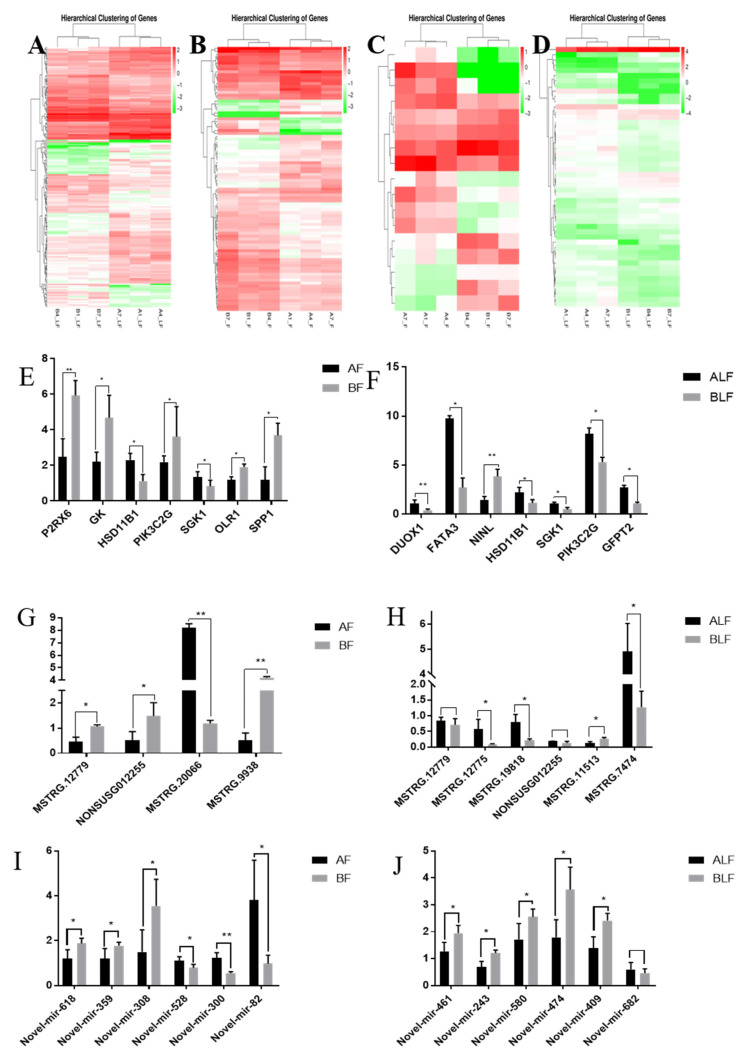
Hierarchical clustering analysis was performed based on the FPKM values of differentially expressed genes under different experimental conditions. (**A**). Cluster analysis of gene levels in castrated and intact pig backfat tissues. (**B**). Cluster analysis of gene levels in castrated and intact pig abdominal tissues. (**C**). Cluster analysis of lncRNA levels in castrated and intact pig backfat tissues. (**D**). Cluster analysis of lncRNA levels in castrated and intact pig abdominal tissues. Quantitative PCR validation. Differentially expressed genes (**E**,**F**), lncRNAs (**G**,**H**) and miRNAs (**I**,**J**) were confirmed by quantitative PCR. The results are shown as the means ± standard deviation of triplicate measurements. AF indicates intact backfat, BF indicates castrated backfat, ALF indicates intact abdominal fat, BLF indicates castrated abdominal fat. * indicates *p* < 0.05, ** indicates *p* < 0.01.

A total of 90 DEGs were identified (Supplementary Materials File S2 Table S4) in the backfat, including 51 upregulated and 39 downregulated (Figure 2A), which were found to have more than 2-fold differential expression between the castrated and intact male pigs (*p* < 0.05). The largest difference was exhibited by PDE12 (phosphodiesterase 12) (log2-fold change = 5.27).

Nine annotated lncRNAs and eight novel lncRNAs had significantly different levels of expression in backfat between castrated and intact male pigs (Supplementary Materials File S2 Table S5), with more than 2-fold differential expression between the castrated and intact male pigs (*p* < 0.05). Compared with castrated pigs, there were eight lncRNAs expressed at higher levels and nine expressed at lower levels in intact male pigs (Figure 2C). Several DE lncRNAs were specifically expressed in intact pig backfat tissue, such as MSTRG.19943, MSTRG.13164 and NONSUSG000058.1, in castrated pigs, such as NONSUSG000375.1, NONSUSG000597.1 and MSTRG.12779.

A total of 20 known miRNAs and 349 novel miRNAs had significantly different levels of expression in backfat between castrated and intact male pigs (Supplementary Materials File S2 Table S6). A total of 144 miRNAs (7 known and 137 novel) were expressed at lower levels, and 225 miRNAs (13 known and 212 novel) were expressed at higher levels in castrated pig backfat than in intact pig backfat.

In abdominal fat, a total of 188 DEGs were identified (Supplementary Materials File S2 Table S7), including 81 upregulated and 107 downregulated genes (Figure 2B), with more than 2-fold differential expression between the castrated and intact male pigs (*p* < 0.05).

Twenty-eight annotated lncRNAs and 22 novel lncRNAs had significantly different levels of expression in abdominal fat tissue between castrated and intact male pigs (Supplementary Materials File S2 Table S8) and were found to have more than 2-fold differential expression between the castrated and intact male pigs (*p* < 0.05). In castrated male pigs, 20 lncRNAs were expressed at higher levels, and 30 lncRNAs were expressed at lower levels than in intact male pigs (Figure 2D). Several DE lncRNAs were specifically expressed in intact pig backfat tissue, such as MSTRG.19943, NONSUSG004879.1, MSTRG.13438, NONSUSG000360.1, NONSUSG015834.1, MSTRG.20051 and MSTRG.20148, or in castrated pigs, such as NONSUSG018491.1, NONSUSG010573.1, NONSUSG001288.1, and MSTRG.12779. A total of 32 known miRNAs and 338 novel miRNAs had significantly different levels of expression in the abdomen between castrated and intact male pigs (Supplementary Materials File S2 Table S9). A total of 157 miRNAs (14 known and 143 novel) were expressed at lower levels, and 213 miRNAs (18 known and 195 novel) were expressed at higher levels in castrated pig abdominal fat tissue than in intact pig abdominal fat tissue.

To verify our RNA-seq data, we randomly selected DEGs, DELs and DEMs for qRT–PCR analysis (Figure 2E–J). The results showed that the differentially expressed RNAs had the same gene expression trends in qRT–PCR and RNA-seq.

### 3.3. Functional Analysis of Differentially Expressed Transcripts

In the backfat, a total of 147 pathways were enriched (Supplementary Materials File S2 Table S10), seven of which were related to fat production and hormone synthesis metabolism. These include the p53 signaling pathway, thyroid hormone signaling pathway, inositol phosphate metabolism, PI3K-Akt signaling pathway, metabolism of xenobiotics by cytochrome P450, ECM-receptor interaction, and PPAR signaling pathway. These pathways have been reported to be associated with fat deposition. These pathways include several key genes, such as CYP1B1, PIK3C2G, COL13A1, OLR1, and GK (Supplementary Materials File S1 Table S4).

**Figure 3:**
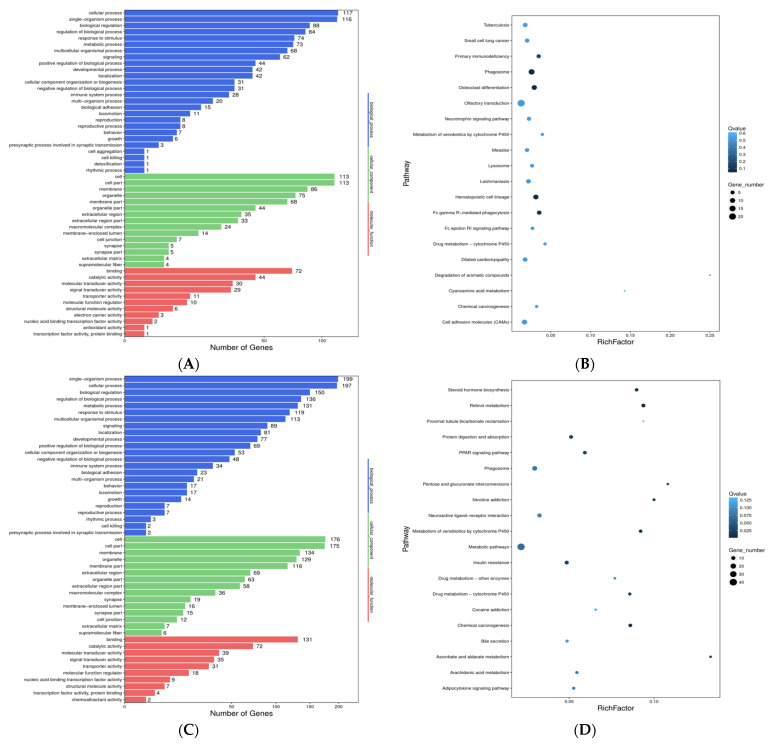

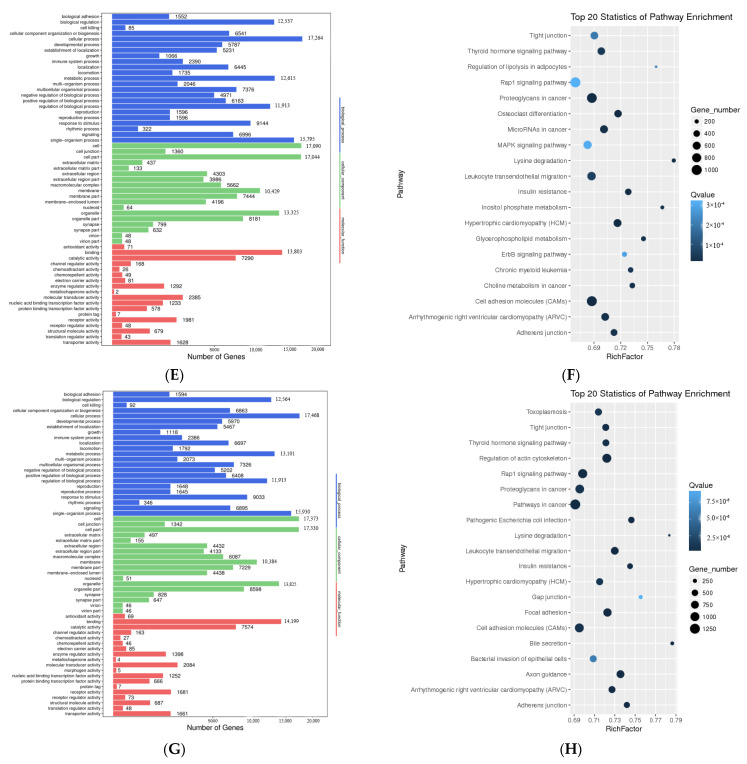
Crucial pathways were clustered from DE lncRNA trans-related genes. (**A**). Gene ontology (GO) function of differentially expressed lncRNA-related mRNAs was classified and analyzed according to trans effect analysis in castrated and intact pig backfat tissues. (**B**). KEGG enrichment analyzed according to trans effect analysis in castrated and intact pig backfat tissues. (**C**). Gene ontology (GO) function of differentially expressed known DEmiRNA target mRNAs in castrated and intact pig abdominal tissues. (**D**). KEGG enrichment analysis according to known DEmiRNA target mRNAs in castrated and intact pig abdominal tissues. Gene ontology (GO) function was classified, and KEGG enrichment was analyzed according to related mRNAs. (**E**). Gene ontology (GO) function of differentially expressed lncRNA-related mRNAs was classified and analyzed according to trans effect analysis inS castrated and intact pig abdominal tissues. (**F**). KEGG enrichment analyzed according to trans effect analysis in castrated and intact pig abdominal tissues. (**G**). Gene ontology (GO) function of differentially expressed known DEmiRNA target mRNAs in castrated and intact pig abdominal tissues. (**H**). KEGG enrichment analysis according to known DEmiRNA target mRNAs in castrated and intact pig abdominal tissues.

A total of 215 genes were predicted to be targets of 16 DE lncRNAs, including 8 cis and 207 trans target genes. GO analysis suggested that target genes are mostly involved in the regulation of biological, metabolic and cellular processes (Figure 3A). KEGG pathway analysis indicated that the genes targeted by DE lncRNAs were involved in pathways related to endocrine and metabolic diseases, lipid metabolism, and energy metabolism (Figure 3B). In addition, further analysis showed that the 215 genes were mainly involved in the immune system, sensory system, signal transduction, signaling molecules, interaction and metabolic pathways.

A total of 72,991 target genes were identified, of which 27,816 and 45,175 genes were predicted to be targets of 20 known miRNAs and 349 novel miRNAs, respectively. GO analysis suggests that known DE miRNAs target the genes that are predominantly involved in biological processes, such as biological adhesion, biological regulation, cell killing, cellular component organization or biogenesis and cellular process (Figure 3C). Glycerophospholipid metabolism, insulin resistance and the thyroid hormone signaling pathway were significantly enriched in the KEGG analysis (Figure 3D).

In abdominal fat, a total of 196 pathways were enriched, nine of which were related to fat production and hormone synthesis metabolism (Supplementary Materials File S2 Table S11), such as the adipocytokine signaling pathway, insulin resistance, calcium signaling pathway, pyruvate metabolism, AMPK signaling pathway, PI3K-Akt signaling pathway, ECM-receptor interaction, metabolism of xenobiotics by cytochrome P450, and PPAR signaling pathway. These pathways have been reported to be related to fat production and hormone synthesis and decomposition. These pathways include several key genes, such as HSD11B1, PCK1, SLC2A4, LEP, and SGK1 (Supplementary Materials File S1 Table S5).

A total of 348 genes were predicted to be targets of 50 DE lncRNAs, including 24 cis and 328 trans target genes (four were shared). GO analysis suggested that cis and trans target genes are mostly involved in the regulation of biological, metabolic and cellular processes (Figure 3E). KEGG pathway analysis indicated that the genes targeted by DE lncRNAs were involved in pathways related to signal transduction, global and overview maps, signaling molecules and interactions, and the endocrine system (Figure 3F). In addition, our further analysis showed that 328 genes were mainly involved in the immune system, sensory system, signal transduction, signaling molecules, interaction and metabolic pathways.

A total of 73,255 target genes were identified, of which 28,100 and 45,155 genes were predicted to be targets of 32 known miRNAs and 338 novel miRNAs, respectively. GO analysis suggests that DE known miRNAs target the genes that are predominantly involved in biological processes, such as biological adhesion, biological regulation, cell killing, cellular component organization or biogenesis and cellular process (Figure 3H). Insulin resistance, the thyroid hormone signaling pathway and bile secretion were significantly enriched in the KEGG analysis (Figure 3I).

### 3.4. lncRNA–miRNA–mRNA Network Construction and Visualization

**Figure 4:**
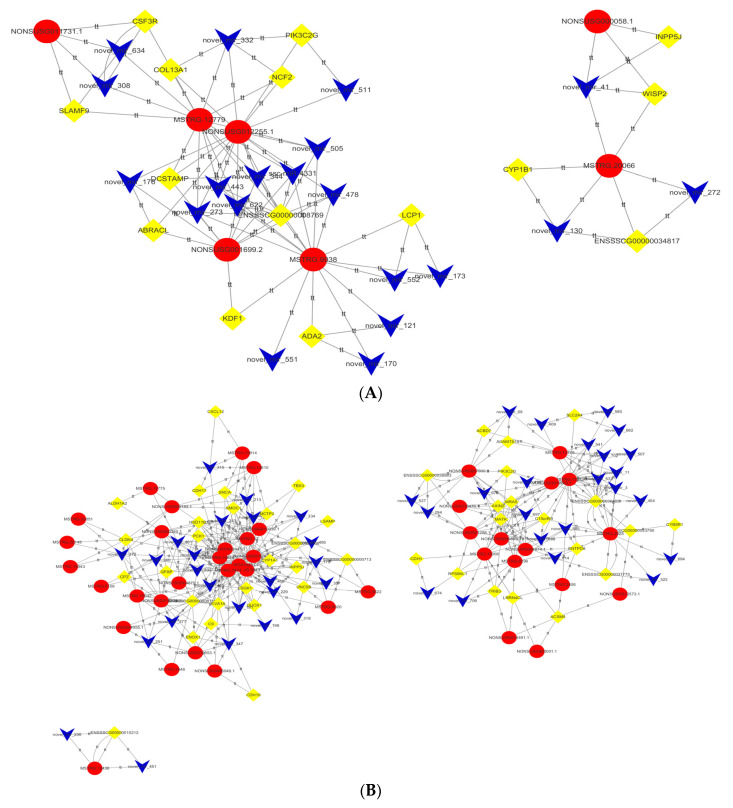
Construction of the ceRNA network. (**A**). The ceRNA network is composed of miRDElncRNAs, DEmiRNAs and DEmRNAs from castrated and intact pig back adipose tissue. (**B**). The ceRNA network is composed of miRDElncRNAs, DEmiRNAs and DEmRNAs from castrated and intact pig adipose tissue. *Circle indicates DE lncRNAs, rhombus indicates DE mRNAs, V shape indicates DE miRNAs*.

In the backfat, the network shows possible interactions among 7 DE lncRNAs, 19 DE miRNAs and 12 DE mRNAs (Figure 4A, Supplementary Materials File S2 Table S12). For example, COL13A1, PIK3C2G, MSTRG.12779, and NONSUSG012255.1 can form a ceRNA network through novel_miR_332, and CYP1B1 and MSTRG.20066 can form a ceRNA network through novel_miR_130.

In abdominal fat, the network shows possible interactions among 40 DE lncRNAs, 47 DE miRNAs and 40 DE genes (Figure 4B, Supplementary Materials File S2 Table S13). Novel_miR_461, novel_miR_474, and novel_miR_243 linked HSD11B1, SGK1, PCK1 and the corresponding lncRNAs, such as MSTRG.19818, NONSUSG010039.1, NON-SUSG003945.1, NONSUSG000360.1, NONSUSG014231.1, MSTRG.32, MSTRG.7474, NONSUSG004879.1, NONSUSG016168.1, MSTRG.12775, and MSTRG.11513.

**Figure 5:**
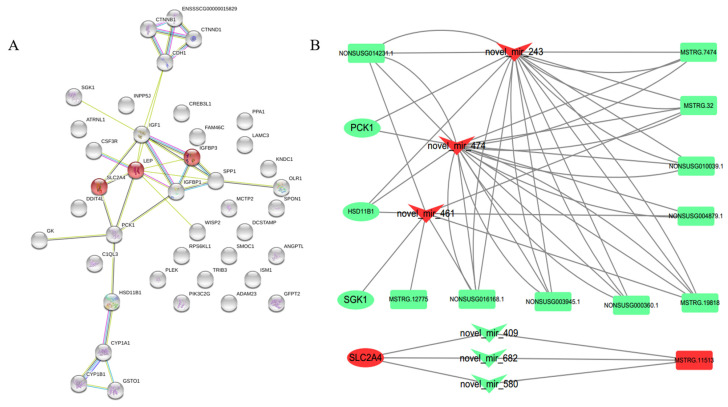
(**A**). Some of the differentially expressed genes were analyzed by protein interactions with STRING. (**B**). LncRNAs regulate the ceRNA network of related genes through miRNAs. *Circle indicates DE mRNAs, rectangle indicates DE lncRNAs, V shape indicates DE miRNAs*.

Among these studies, in backfat, genes (such as COL13A1 and PIK3C2G) and pathways (PI3K-Akt signaling pathway, metabolism of xenobiotics by cytochrome P450, ECM-receptor interaction, etc.) were identified to play roles in signaling pathways involved in cell proliferation, cell survival, cell migration, and intracellular protein trafficking. In abdominal fat, common genes (such as HSD11B1, PCK1, SLC2A4 and SGK1) and pathways (insulin resistance, adipocytokine signaling pathway, etc.) were identified which participate in the regulation of fat deposition and androgen metabolism. We analyzed the protein interaction relationships among these genes through STRING (Figure 5A) and found that they had protein interactions with each other. At the same time, through the construction of the ceRNA network, we found that these genes could build an interrelated network relationship through common miRNAs and lncRNAs (Figure 5B).

### 3.5. Double Fluorescence Binding Verification Experiment

**Figure 6:**
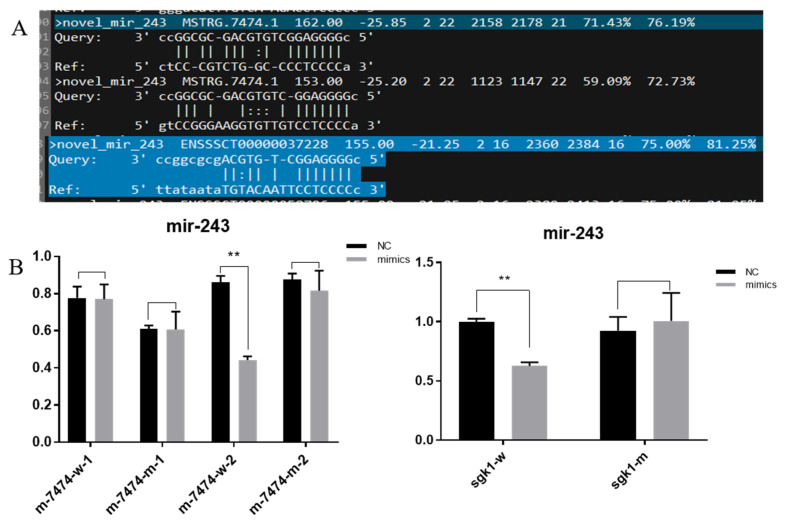
(**A**). MiRNA binding locations predicted by miRanda software. (**B**). Double fluorescence verification experiment. ** indicates *p* < 0.01, Nc is the control group, mimics refers to the overexpression of miR-243.

Through a ceRNA network, we selected novel-miR-243, M-7474 and SGK1 to verify their interaction. The results of the double luciferase reporter gene detection system showed that SGK1 and M-7474 mutant vector plasmids were transfected and cloned. In the experiment, the novel-miR-243 group was compared with the negative control group, and there was no significant difference in the luciferase activity (*p* > 0.05). In the transfection experiment with the SGK1 and M-7474 vector plasmids, the luciferase activity of the novel-miR-243 group was significantly inhibited, and there was a significant difference in luciferase activity compared with the negative control group (*p* < 0.05). Novel-miR-243 and M-7474 were predicted to have two binding sites, but only the second site could bind after our double fluorescence verification. The predicted binding position and dual fluorescence results are shown in Figure 6.

### 3.6. Functional Verification of Overexpressed M-7474

**Figure 7:**
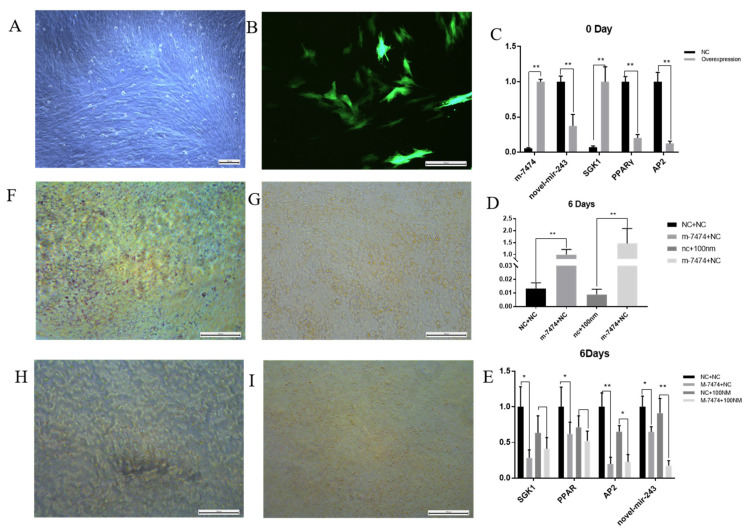
Preadipocyte differentiation experiment of the M-7474-overexpressing group. (**A**). Bright field image of preadipocytes overexpressing M-7474. (**B**). Fluorescent image of the anterior adipocytes after overexpression of M-7474. (**C**–**E**). qPCR quantification of related genes. (**F**). Oil red O staining was performed 6 days after differentiation in the NC group without testosterone addition. (**G**). Oil red O staining was performed 6 days after differentiation in the overexpressed M-7474 group without testosterone addition (**H**). The NC group was differentiated with 100 nm testosterone for 6 days after oil red O staining (**I**). The overexpressed M-7474 group was differentiated with 100 nm testosterone for 6 days after oil red O staining. NC indicates control group, M-7474 indicates lncRNA overexpression group, Nc + 100 nM indicates NC group was added with 100 nm testosterone, M-7474 + NC indicates lncRNA overexpression group was added with 100 nm testosterone. * indicates p < 0.05, ** indicates p < 0.01.

To investigate the potential role of M-7474 in porcine preadipocyte differentiation, m-7474-overexpressing preadipocytes were constructed (Figure 7A,B). At the same time, the expression levels of SGK1, novel-miR-243 and other genes in M-7474-overexpressing preadipocytes were determined. When preadipocytes were undifferentiated, novel-miR-243 expression was decreased and SGK1 was increased after overexpression of M-7474 (Figure 7C), which is consistent with the ceRNA relationship. We found a significant reduction in the expression of adipogenic differentiation markers in the M-7474 overexpression group by RT-qPCR at day 6 after the induction of differentiation, and the oil red results showed that the lipid droplet content was also reduced. Treatment with the addition of 100 nM testosterone also resulted in a significant increase in M-7474, which similarly inhibited adipogenesis. The expression of novel-miR-243 decreased with the overexpression of M-7474. However, we also found that the expression of SGK1, a target gene of novel-miR-243, decreased in the group overexpressing M-7474 as well as in the group supplemented with 100 nM testosterone during differentiation (Figure 7D–I).

## 4. Discussion

This study analyzed, for the first time, the expression of lncRNAs, miRNAs and mRNAs in the backfat and abdominal fat tissues of intact and castrated male pigs. The aim was to identify potential lncRNA-miRNA–mRNAmiR networks related to adipogenic differentiation and lipid metabolism after castration. The castration of domestic pigs has an important effect on the accumulation of adipose tissues [44]. Recently, an increasing number of noncoding ribonucleic acids, including microribonucleic acid, cyclic ribonucleic acid and cyclic ribonucleic acid, have been proven to serve important regulatory roles in gene expression networks affecting many biological processes or diseases [45,46]. Noncoding RNAs (ncRNAs) have evolved into epigenetic regulators of gene expression in eukaryotes. MiRNAs and lncRNAs are the most abundant regulatory noncoding RNAs. Every ncRNA class regulates gene expression by different mechanisms. LncRNAs, miRNAs and mRNAs form a large-scale ceRNA crosstalk network through MREs, which is of exciting significance for posttranscriptional gene regulation in various physiological and pathophysiological processes [47,48]. Long noncoding RNAs can regulate protein-coding gene expression at both the transcriptional and posttranscriptional levels [49]. Among these differentially expressed miRNAs (DE miRNAs), miR-122 [50], miR-4331 [51], miR-124a [52,53], miR-206 [54], miR-224 [55] and miR-129a [56] have been reported to be involved in lipid metabolism, obesity and metabolic diseases.

Some studies have shown that visceral fat contains higher levels of adrenocorticosteroids and androgen receptors and has higher catabolic activity, insulin resistance and glucose uptake capacity than subcutaneous fat and is more sensitive to adrenosteroids [15,16]. Abdominal fat is more prone to insulin resistance than is backfat [57]. The distribution of glucocorticoid receptors in visceral adipose tissue is significantly higher than that in subcutaneous adipose tissue [14], which makes glucocorticoids more effective in visceral adipose tissue. Activating glucocorticoid receptors promotes the differentiation of visceral adipocytes and lipid accumulation and increases the viscera distribution of fat. In addition, Starr et al. [58] showed that under obesity, chemokines secreted by adipocytes activated and attracted various inflammatory cells into adipose tissue, which led to chronic low-grade inflammation of the adipose tissue. This is consistent with the significantly increased expression of inflammatory marker genes in adipose tissues after castration in our study, which is especially true of abdominal fat. For example, SPP1 (secreted phosphoprotein 1) is highly expressed in the abdominal fat of castrated pigs and is a cytokine that upregulates the expression of interferon-gamma and interleukin-12 [59].

ECM-receptor interactions play an important role in tissue and organ morphogenesis and the maintenance of cell and tissue structure and function. These interactions lead to direct or indirect control of cellular activities such as adhesion, migration, differentiation, proliferation, and apoptosis [60]. By analyzing the enrichment of differential RNA, miRNA and lncRNA and constructing the ceRNA network, we found that the effects of testosterone on backfat adipocytes are more related to adipocyte proliferation, differentiation, migration, apoptosis and signal transduction. Adipose tissue can be used as an endocrine organ to secrete adipokines to regulate glucose balance in the body, and leptin (*LEP*) can regulate glucose and lipid metabolism, which can be regarded as a marker of insulin resistance [61]. In our study, LEP was highly expressed in the abdominal adipose tissue of castrated pigs. These results indicated that insulin resistance in castrated pigs affected abdominal fat deposition. The insulin resistance signaling pathway is closely related to diabetes, metabolic syndrome and obesity and is also closely related to the activities of phosphoenolpyruvate carboxykinase 1 (PCK1) and solute carrier family 2 member 4/GLUT4 (SLC2A4) [62,63]. The effect of testosterone on abdominal fat cells is more closely related to the effect of hormones on cells, the metabolism of lipid-related products and the production of inflammatory factors. Our study has shown that testosterone-induced fat deposition works differently in different body parts, with a greater effect on abdominal fat deposition, which coincides with the results of a previous study.

Testosterone can bind to the androgen receptor and activate gene expression in the nucleus [64], which is considered the main pathway for testosterone to exercise its function. However, some new studies have also found that there are two pathways through which testosterone function is exercised, one being androgen receptor dependent and the other being nonandrogen receptor dependent [65,66]. After inhibiting the androgen receptor, testosterone can still exercise its related functions through other metabolic pathways, which show extensive diversity [67]. Pregnenolone, synthesized from cholesterol, is the precursor for the synthesis of testosterone, estradiol, and cortisol [68]. Testosterone can be converted into estradiol in peripheral tissues by the action of P450-related genes in the aromatization signaling pathway [69]. Cortisol, which is converted from pregnenolone, is one of the most potent adrenocortical hormones affecting carbohydrate metabolism [70]. Therefore, testosterone may affect body fat changes by affecting changes in cortisol content. Cortisol can be converted by HSD11B1 (11β-hydroxysteroid dehydrogenase type 1, 11β-HSD1) to inactive corticosterone [71]. It can promote the occurrence and development of insulin resistance and obesity by increasing intracellular glucocorticoid levels [72]. Studies by London and Castonguay [73] have shown that 11β-HSD1 is more active in rodent omentum adipose tissue. HSD11B1 is closely related to the accumulation and metabolic disorders of abdominal fat [74,75,76]. SGK1 (serum and glucocorticoid-induced protein kinase 1) is regulated by glucocorticoids and is involved in the development of obesity [77]. It has been proposed that SGK1 promotes obesity by stimulating the Na+ glucose cotransporter SGLT1 (solute carrier family 5 member 1). SGLT1 can accelerate the intestinal uptake of glucose [78]. The rapid absorption of glucose in the intestinal tract leads to excessive release of insulin and fat deposition, followed by a reduction in blood glucose concentration, resulting in repeated glucose uptake and eventually leading to obesity [79]. SGK1 promotes glucose transporter membrane abundance via SLC2A4 phosphorylation at Ser274. In unstimulated muscle cells and adipocytes, SLC2A4 is distributed within vesicles in the cytoplasm and is unable to function. After insulin stimulation, the activated insulin receptors phosphorylate a variety of proteins in the cells and then trigger the translocation of SLC2A4 vesicles to the cell membrane and their fusion with the cell membrane to increase glucose uptake [80,81]. After the stimulation disappeared, the cells transported SLC2A4 from the extracellular membrane to the cells through endocytosis and stored it in vesicles [82]. Thus, SGK1 may contribute to the insulin- and SLC2A4-dependent regulation of cellular glucose uptake [83]. In our study, the low expression of HSD11B1 in abdominal fat after castration suggests its reduced ability to participate in the conversion of cortisol to corticosterone, which in turn enables enhanced glucocorticoid activity in adipose tissue. HSD11B1, including its ceRNA network, plays an important role in the involvement of testosterone in abdominal fat accumulation by regulating the interconversion between cortisol and corticosterone. However, in our study, the expression level of SGK1 in the castrated group was low, which was inconsistent with the existing research. We speculate that the change in the expression level may be related to the stage and other regulatory factors. The expression level of SLC2A4 is consistent with the high expression in castrated pigs, which is consistent with the promotion of glucose absorption and thus the increase in fat deposition. In other words, after castration, the transformation of androgen and estradiol was regulated through the cytochrome P450 signaling pathway, the activity of glucocorticoids was regulated by changing HSD11B1 expression, and the expression of SGK1 was regulated by glucocorticoid activity, which in turn regulated the expression of SLC2A4. This ultimately affects glucose uptake by adipocytes and leads to obesity.

PCK1 is a rate-limiting enzyme that can regulate gluconeogenesis and participate in maintaining blood glucose levels [84,85,86]. There are multiple cytokine pathways by which inflammation inhibits PEPCK expression in adipose tissue, which could contribute to the increased mobilization of fatty acids during inflammation [87]. A large number of inflammatory cytokines are produced in the process of obesity caused by castration. There are multiple cytokine pathways by which inflammation inhibits PEPCK expression in adipose tissue, which could contribute to the increased mobilization of fatty acids during inflammation [87]. In our study, the expression of PCK1 was lower in the castration group, which indicates that adipose tissue lipolysis is markedly stimulated during inflammation and that the expression of PCK1 in adipose tissue, which is essential for glyceroneogenesis, would also be markedly suppressed to enhance the release of free fatty acids.

Our data found that testosterone could promote the expression of M-7474 to inhibit preadipocyte differentiation during preadipocyte differentiation, and overexpression of M-7474 could also inhibit preadipocyte differentiation. RT-qPCR revealed a ceRNA relationship between M-7474 and novel-miR-243 as well as SGK1 in preadipocytes. Based on our analysis of the data, we concluded that castrated pigs are more likely to suffer from insulin resistance and metabolic syndrome, especially in abdominal fat, and that the effect of castration on backfat adipocytes is more related to cell migration, differentiation and apoptosis. The occurrence of insulin resistance and metabolic syndrome in the body, in turn, changes the expression of these genes and their regulatory factors, which further intensifies the increase in inflammatory factors and fat in the body and places the body in a pathological state. At the same time, the constructed ceRNA network indicated that after castration, the transformation of testosterone and estradiol was regulated through the cytochrome P450 signaling pathway, the activity of glucocorticoids was regulated by changing HSD11B1 expression, and the expression of SGK1 was regulated by glucocorticoid activity, which in turn regulated the expression of SLC2A4. In this regulatory process, lncRNAs such as M-7474 and corresponding miRNAs such as novel-miR-243 may play important roles. This ultimately affects glucose uptake by adipocytes and leads to obesity. In this study, testosterone was identified as the most important factor affecting fat deposition after castration of boars. Studies have shown that testosterone can significantly inhibit the production of fat [88,89], and testosterone is the most abundant androgen produced by the testes of boars from fetal period to puberty [90,91]. Leydig cells of the boar testis are the main site of testosterone production, however, other hormones such as the 16-unsaturated steroids, the oestrogens and dehydroepiandrosterone (DHEA) can also be produced [92]. These hormones may also have important effects on lipid metabolism in barrows, their specific effects remain to be further verified in the future experiments.

## 5. Conclusions

In this study, we established a pig model with obesity due to androgen deficiency and compared it with normal pigs to screen genes that affect fat deposition. According to the GO and KEGG results, the differentially expressed genes in backfat were mainly involved in cell proliferation, differentiation, migration, apoptosis, signaling and other related pathways. Abdominal fat differential genes are more often associated with hormonal influences on cells, metabolism of lipid-related products, and generation of inflammatory factors. Our study suggests that the effects of androgen on adipose tissues in different body parts are inconsistent, and that abdominal fat is more vulnerable to testosterone effects than is backfat. Castration can affect the migration, differentiation and apoptosis of backfat adipocytes through COL13A1, PIK3C2G and their regulatory factors. Castration further leads to glycolipid metabolic disorders and increased expression of inflammatory cytokines by affecting the expression of HSD11B1, SGK1, PCK1, and SLC2A4 in abdominal adipose tissues and their regulatory factors. LncRNAs such as M-7474 and corresponding miRNAs such as novel-miR-243 may play an important role in testosterone regulation of fat deposition in pigs, which affects meat quality as well as pig production benefits.

## Data Availability

All RNA-seq data were deposited in the Gene Expression Omnibus under accession number PRJNA801764.

## Supplementary Materials

The following are available online at https://www.mdpi.com/article/10.3390/genes13040668/s1, Figure S1: The left side shows the back fat and suet of the castrated pigs, and the right side shows the back fat and suet of the intact pigs, Figure S2: The Pearson correlation values, Figure S3: Table S1: Sample collection information, Table S2: Primer sequences used in qRT-PCR analysis, Table S3: Statistical summary for RNA sequencing results, Table S4: Significantly enriched GO terms and KEGG pathways related to backfat generation, Table S5: Significantly enriched GO terms and KEGG pathways related to abdominal fat generation, Table S6: Differentially expressed mirnas in backfat tissue, Table S7: MRNA differentially expressed in abdominal adipose tissue, Table S8: LncRNA differentially expressed in abdominal adipose tissue, Table S9: MiRNA differentially expressed in abdominal adipose tissue, Table S10: KEGG enrichment pathway of differential genes of backfat in castrated and intact pigs, Table S11: S11 KEGG enrichment pathway of differential genes of abdominal adipose tissue in castrated and intact pigs, Table S12: lncRNA–miRNA–mRNA Network Construction in backfat tissue, Table S13: lncRNA–miRNA–mRNA Network Construction in abdominal adipose tissue.
