# Supplementary material for: Integrated Analysis of the ceRNA Network and M-7474 Function in Testosterone-Mediated Fat Deposition in Pigs
Source: Genes (Basel). 2022 Apr 10;13(4):668. doi: 10.3390/genes13040668 (PMC9032878; doi:10.3390/genes13040668)
Supplement: Supplementary file 1 [file genes-13-00668-s001.zip › Additional fileS1.Table S1.pdf]

Table S1.Sample collection information

	**A1(intact)**	**B1(castration)**	**A4(intact)**	**B4(castration)**	**A7(intact)**	**B7(castration)**
Live weight（Kg）	150	140.5	166.5	154	134	112
carcass weight（Kg）	112.6	107.9	128.2	119.2	100	85.7
First back fat thick（mm）	37.76	44.90	34.66	48.73	39.63	47.25
Finally back fat thick（mm）	12.04	30.040	16.79	25.25	9.43	25.01
Finally lumbar backfat thickness（mm）	8.99	22.98	9.93	22.38	9.41	19.03
leaf fat（Kg）	0.494	1.528	0.742	1.114	0.338	0.828
