# Supplementary material for: Integrated Analysis of the ceRNA Network and M-7474 Function in Testosterone-Mediated Fat Deposition in Pigs
Source: Genes (Basel). 2022 Apr 10;13(4):668. doi: 10.3390/genes13040668 (PMC9032878; doi:10.3390/genes13040668)
Supplement: Supplementary file 1 [file genes-13-00668-s001.zip › Additional fileS1.Table S2.pdf]

Table S2 Primer sequences used in qRT-PCR analysis

Gene name	Primer	Amplicon Length(bp)
MSTRG.12779	F:5'GCTGTAGCTCACGGACAGAG3'	
	R:5'TGTTCTCCACATGGACCGAC3'	255
MSTRG.14129	F:5'ATCGAGTCCCAATCACCCAC3'	
	R:5'CTGGTGATTTGCAGCCCTTG3'	197
MSTRG.20066	F:5'CCCTGAATGCCCCTTACTGG3'	
	R:5'ACCTGACCCTGGCCTAATGT3'	157
MSTRG.12775	F:5'GGCCAATTGAGGACCCAAGA3'	
	R:5'AGTTGTCCCTCTCACCACCT3'	263
MSTRG.19818	F:5'ACAACTAGCCACCTGTTCGG3'	
	R:5'GGCCTTAAAGGGGTCCTGTC3'	255
MSTRG.11513	F:5'AGCATTCTGTGATGCAGCCT3'	
	R:5'GCACCAAATACTCACCACGC3'	134
MSTRG.7474	F:5'GCATCATGTGGGGTTGCTTG3'	
	R:5'TCTAAAAGCGAGGCCGAGTC3'	207
MSTRG.9938	F:5'TGTCCAGGTTGTATGGCTCA3'	
	R:5'CAGGCTGGAGTCAGGGAAAT3'	147
NINL	F:5'CTGTCCCGCCAAAGTATGT3'	
	TTCCCGAAGACCTCACTCC	262
CLDN11	CCATTCTCCTGCTGCTGACA	
	TGCATACAGGGAGTAGCCAAAG	197
DUOX1B	TTGGGACTCTGTGCTGCTTC	
	CATGATACTCTGGCGGTTCTG	113
P2RX6	CTCGCCAAGAAAGGCTACCAG	133

	GCTTCACGAAATCAGCCACATC	
PIK3C2G	F: 5' GCAATGAGCTTCTGAGCTTGC 3'<br>R: 5' CGGAGATTGCTGTCCTGTGT 3'	127
SGK1	F:5'GAACACAACGGCACGACATCC3'<br>R:5'TGGAGGCAGACCATACAGCATC3'	144
FAT3	F:5'TCCTGAAGATTGTGGACGGC3'<br>R:5'AGGGCTCCGAAGTAGATGGT3'	232
PPARγ	F:5'CTTATTGACCCAGAAAGCGATG3'<br>R:5'GTCTTCATAGTGTGGAGTGGAA3'	213
GFPT2	F: CAGGGTCGCCCGATTAT<br>R: CACGCTCTTGTTCTCATTCC	223
SPP1	F: ACAGCCGCATCAGCATTG<br>R: AAGTCGTCCGTTTCCTCC	311
HSD11B1	F:5'TGGCGAATCCACTTGTT3'<br>R:5'GGTTGTACGTTGAGTATCCC3'	181
OLR1	F:5'GCGGCAAACTTTTCAGGTCC3'<br>R:5'ACTGGAATGGGCGATGGTTT3'	189
β-actin	F:5'AGAGCAAGAGAGGCATCCTG3'<br>R:5'CACGCAGCTCGTTGTAGAAG3'	111
novel_mir_618	F:5'GTGAAATGTTTAGGACCACT3'	
novel_mir_359	F:5'TGTGGTGCCTGACGTCTTGG3'	
novel_mir_528	F:5'AGGACGGGAAGAGAGGAGGGCGT3'	
novel_mir_308	F:5'CGGCTCTGGGTCTGTGGGGAG3'	
novel_mir_300	F:5'TGCGTGGCTTCTCTGTGCAGTC3'	
novel_mir_82	F:5'AAATCTCTGCAGGCAAATGTGA3'	
novel_mir_461	F:5'TGGAGGAGGAATGGGGAGGACA3'	

novel_mir_474	F:5'TGGGGAGGGCCGGACAGAGC3'
novel_mir_243	F:5'CGGGGAGGCTGTGCAGCGCGGC3'
novel_mir_580	F:5'AGAGGGGGTATAGCTCAGTGG3'
novel_mir_409	F:5'AACGGAGGCCTCTTACCAGC3'
novel_mir_682	F:5'AGGCCAGCGAGTAGAGAAGC3'
U6	F:5'GCTTCGGCAGCACATATACT3'
