# Supplementary material for: Integrated Analysis of the ceRNA Network and M-7474 Function in Testosterone-Mediated Fat Deposition in Pigs
Source: Genes (Basel). 2022 Apr 10;13(4):668. doi: 10.3390/genes13040668 (PMC9032878; doi:10.3390/genes13040668)
Supplement: Supplementary file 1 [file genes-13-00668-s001.zip › Additional fileS1.Table S3.pdf]

Table S3. Statistical summary for RNA sequencing results

Sample	Clean Reads(M)	Clean Bases(G)	Q20(%)	Q30(%)	GC(%)	Total Mapped	Read Length(bp)
A1_F	96.1643M	14.4247G	93.95	86.79	56.21	74690144(77.71%)	150
A1_LF	97.4015M	14.6102G	97.28	93.32	54.37	83827810(86.16%)	150
A4_F	96.4951M	14.4743G	94.47	87.78	55.79	76975996(79.84%)	150
A4_LF	96.2308M	14.4346G	97.09	92.99	53.95	81851802(85.26%)	150
A7_F	97.5291M	14.6294G	97.09	93.05	51.37	83717986(85.90%)	150
A7_LF	97.4236M	14.6135G	96.87	93.01	51.91	82263422(84.65%)	150
B1_F	97.0317M	14.5548G	94	86.99	55.36	74450694(76.78%)	150
B1_LF	97.3676M	14.6051G	96.97	92.68	56.9	82909806(85.32%)	150
B4_F	97.1519M	14.5728G	96.87	92.87	54.16	84613052(87.18%)	150
B4_LF	97.1956M	14.5793G	96.92	92.98	54.87	84139274(86.78%)	150
B7_F	96.0861M	14.4129G	96.99	92.91	50.58	81369440(84.72%)	150
B7_LF	97.0402M	14.5560G	96.84	92.97	52.98	80961378(83.59%)	150
