# Supplementary material for: Integrated Analysis of the ceRNA Network and M-7474 Function in Testosterone-Mediated Fat Deposition in Pigs
Source: Genes (Basel). 2022 Apr 10;13(4):668. doi: 10.3390/genes13040668 (PMC9032878; doi:10.3390/genes13040668)
Supplement: Supplementary file 1 [file genes-13-00668-s001.zip › Additional fileS1.Table S4-S5.pdf]

Table S4. Significantly enriched GO terms and KEGG pathways related to backfat generation

Gene	Gene id	CateGory
CYP1A1	ENSSSCG00000033844	ko00140,ko00380,ko00980,ko04913,ko05204,ko05206
GSTO1	ENSSSCG00000022351	ko00480,ko00980,ko00982,ko05200,ko05204,ko05215
SPP1	ENSSSCG00000009216	ko04015,,ko04145,,ko04151,,ko04360,,ko04380,ko04510,ko04512,ko04514,ko04611,ko0462,ko04640,ko04670,ko04810,ko04919,ko05100,ko05130,ko05131,ko05133,ko05140,ko05145,ko05200,ko05205,ko05206,ko05222,ko05410,ko05412,ko05414
OLR1	ENSSSCG00000000647	ko03320,ko04145
GK	ENSSSCG00000012202	ko00561,ko01100,ko03320
CYP1A1	ENSSSCG00000033844	GO:0016021,GO:0006809,GO:0042572,GO:0030199,GO:0033629,GO:0030336,GO:2000377,GO:0005739,GO:0032088,GO:0046427,GO:0009636,GO:0002930,GO:0071603,GO:0008631,GO:0046466,GO:0070301,GO:0045766,GO:0009404,GO:0019369,GO:0008285,GO:0061304,GO:0071407,GO:0010575,GO:0020037,GO:0042574,GO:0055114,GO:0006805,GO:0043065,GO:0008202,GO:0008210,GO:0005506,GO:0016712,GO:0043542
GSTO1	ENSSSCG00000022351	GO:0070062,GO:0004364,GO:0050610,GO:0005829,GO:0042178,GO:0055114,GO:0006749,GO:0071243,GO:0010880,GO:0019852,GO:0098869,GO:0060315,GO:0060316,GO:0045174
SPP1	ENSSSCG00000009216	GO:0031214,GO:0005125,GO:0001649,GO:0033280,GO:0005615,GO:0050840,GO:0007155,GO:0045780,GO:0070062,
OLR1	ENSSSCG00000000647	GO:0030246,GO:0016021,GO:0005041,GO:0045908,GO:0032930,GO:0005654,GO:0070062,GO:0043235,GO:0005515,GO:0045121,GO:0005886,GO:0006898,GO:0007155,GO:0006954,GO:0002376
GK	ENSSSCG00000012202	GO:0019217,GO:0005829,GO:0005739,GO:0005634,GO:0006071,GO:0042593,GO:0070062,GO:0006641,GO:0046167,GO:0016310,GO:0004370

Table S5. Significantly enriched GO terms and KEGG pathways related to abdominal fat generation

Gene	Gene id	CateGory
PCK1	ENSSSCG00000007507	ko00010,ko00020,ko00620,ko01100,ko03320,ko04068,ko04151,ko04152,ko04910,ko04920,ko04922,ko04931,ko04964
SLC2A4	ENSSSCG00000023915	ko04068,ko04152,ko04910,ko04920,ko04930,ko04931
LEP	ENSSSCG00000040464	ko04060,ko04080,ko04152,ko04630,ko04920,ko04932
SGK1	ENSSSCG00000021443	ko04068,ko04151,ko04960
HSD11B1	ENSSSCG00000015616	ko00140,ko00980,ko01100,ko05204
PCK1	ENSSSCG00000007507	ko00010,ko00020,ko00620,ko01100,ko03320,ko04068,ko04151,ko04152,ko04910,ko04920,ko04922,ko04931,ko04964
SLC2A4	ENSSSCG00000023915	GO:0010021,GO:0005355,GO:0070062,GO:0009897,GO:0030140,GO:0048471,GO:0032869,GO:0042383,GO:0005768,GO:0045121,GO:0016021,GO:0005886,GO:0050873,GO:0032593,GO:0071470,GO:0042593,GO:1904659,GO:0030659,GO:0005887,GO:0005829
LEP	ENSSSCG00000040464	GO:0008203,GO:0032008,GO:0006635,GO:0042269,GO:0032310,GO:0072606,GO:0005179,GO:0021954,GO:0060612,GO:0032868,GO:0006112,GO:0006909,GO:0005615,GO:0042593,GO:0008343,GO:0072604,GO:0030300,GO:0042102,GO:1900745,GO:0001890,GO:0050999,GO:0050810,GO:0032615,GO:0042755,GO:0098868,GO:1900015,GO:0008206,GO:0038108,GO:0030217,GO:0045639,GO:0008083,GO:0042445,GO:0005737,GO:0014068,GO:2000366,GO:0032817,GO:0051897,GO:0050796,GO:0051726,GO:0051428,GO:0010507,GO:0046850,GO:0090335,GO:0006111,GO:0007260,GO:0048639,GO:0019953,GO:1990051,GO:0046325,GO:0000122,GO:0070093,GO:0001936,GO:0002021,GO:0032760,GO:0045765
SGK1	ENSSSCG00000021443	GO:0006974,GO:0005789,GO:0004712,GO:0035091,GO:0001558,GO:0005654,GO:0032411,GO:0006468,GO:0018105,GO:0005524,GO:0042981,GO:0017080,GO:0017081,GO:0005739,GO:0035556,GO:0048812,GO:0042127,GO:0015459,GO:0010765,GO:0005737,GO:0005886,GO:0005246,GO:0006883,GO:0004674
HSD11B1	ENSSSCG00000015616	GO:0005789,GO:0008202,GO:0016021,GO:0003845,GO:0030324,GO:0070524,GO:0055114
