# Supplementary material for: Integrated Analysis of the ceRNA Network and M-7474 Function in Testosterone-Mediated Fat Deposition in Pigs
Source: Genes (Basel). 2022 Apr 10;13(4):668. doi: 10.3390/genes13040668 (PMC9032878; doi:10.3390/genes13040668)
Supplement: Supplementary file 1 [file genes-13-00668-s001.zip › Additional fileS3.Figure S1-S2.pdf]

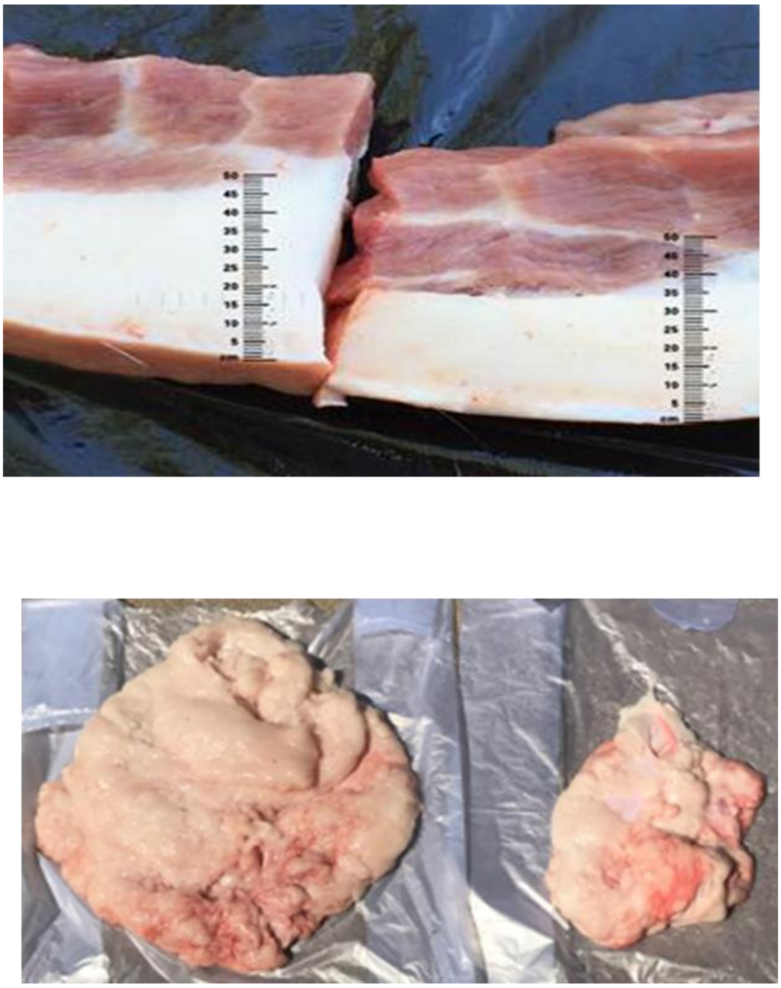

Figure S1.The left side shows the back fat and suet of the castrated pigs, and the right side shows the back fat and suet of the intact pigs

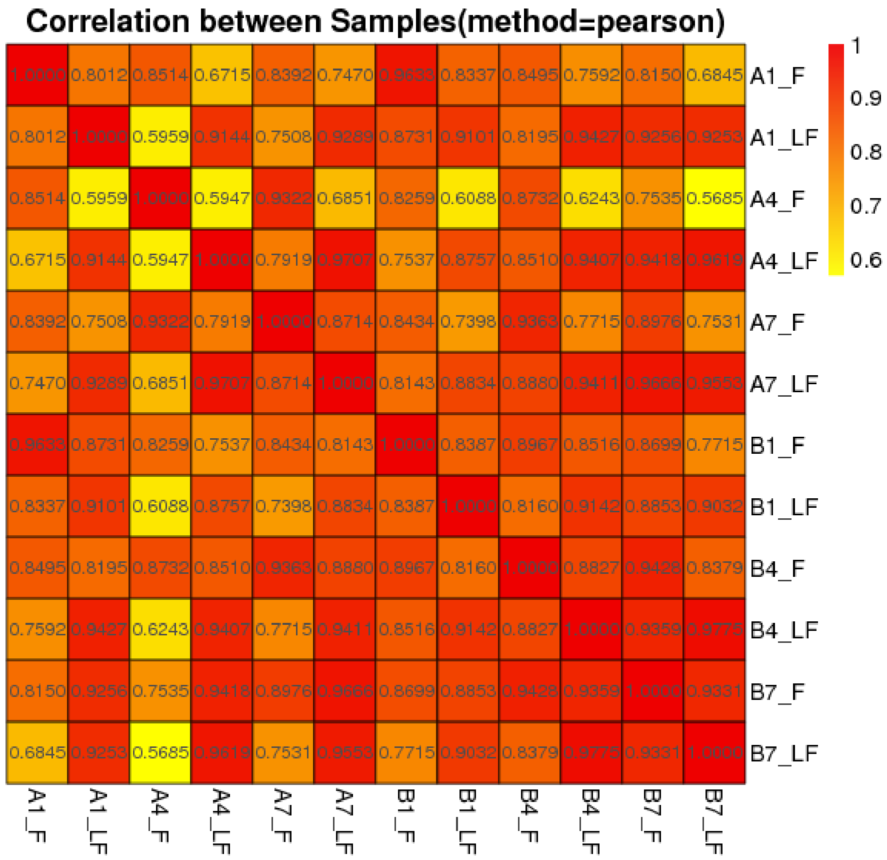

Figure S2 .The Pearson correlation values
